# P-462. Disparities in HPV Vaccination among HIV Patients at a Rural Federally Funded HIV Clinic- A Retrospective Study

**DOI:** 10.1093/ofid/ofae631.661

**Published:** 2025-01-29

**Authors:** Shanthi Reddy Sripathi, Barath Prashanth Sivasubramanian, Madhumithaa Jagannathan, Avinash Javvaji, Dinesh Kumar Shanmugam, Bharath Duraisamy Swami Kannan, Shashvat Joshi, Priyanshu Jain, Sonia Babu, Kathyrn Smith, Rutul Dalal, Raghavendra Tirupathi

**Affiliations:** Osmania Medical College, Hyderabad, Telangana, India, Hyderabad, Telangana, India; University of Texas Health San Antonio, San Antonio, Texas; M.I.M.E.R Medical College, Talegaon Dabhade, Pune, Maharashtra, India, Chambersburg, Pennsylvania; Chalmeda Anandrao Institution of Medical Sciences, Karimnagar, Telangana, India, Chambersburg, Pennsylvania; PSG IMSR, Avinashi Road, Peelamedu, Coimbatore, Tamil Nadu, India, Chambersburg, Pennsylvania; Government Sivagangai Medical College, Sivaganga, Tamil Nadu, India, Chambersburg, Pennsylvania; Shanghai Medical College, Fudan University, Shanghai, China, Chambersburg, Pennsylvania; Kasturba Medical College, Manipal, Udupi, Karnataka, India, Chambersburg, Pennsylvania; Ramaiah Medical College, Bangalore, Karnataka, India, Chambersburg, Pennsylvania; Keystone Health, Chambersburg, Pennsylvania; Medical Director, Penn State Health (Eastern Region), Penn State Health St. Joseph Medical Center, Pennsylvania, USA, Lancaster, Pennsylvania; Keystone Health, Chambersburg, Pennsylvania, USA, Chambersburg, Pennsylvania

## Abstract

**Background:**

Our study aimed to evaluate the demographic factors of HPV vaccination among racial minorities of HIV patients at a non-academic Ryan White clinic in rural Pennsylvania.

**Figure:**
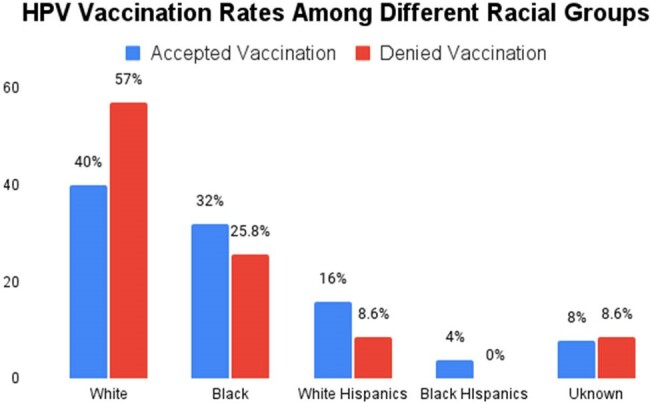

**Methods:**

A retrospective cross sectional study (n=143) was conducted on a population composed of White (51.05%), White Hispanics WH (11.19%), Black (27.97%), Black Hispanics BH (1.4%), and Unknown (8.39%). Proportions and chi-square tests were used and multivariate regression analysis was done with p≤0.05 as significant.

**Figure:**
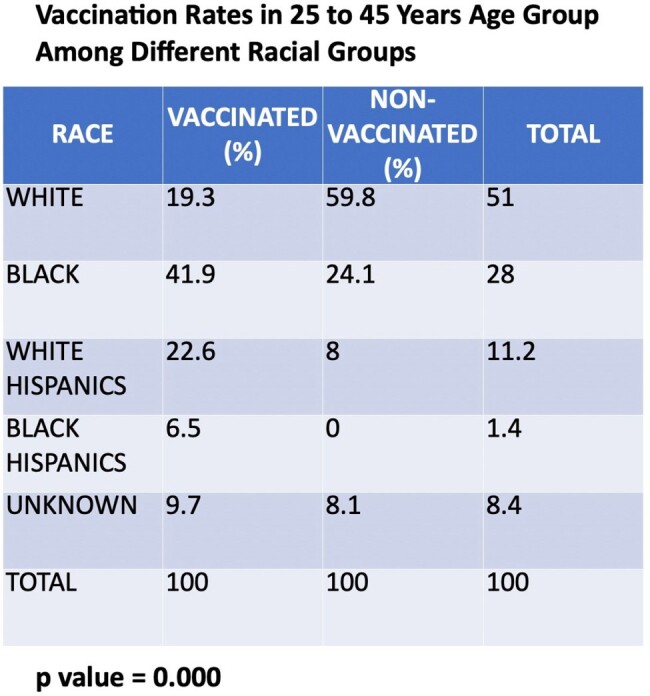

**Results:**

Our study found that 35% of the population was vaccinated, with 60.7% receiving three doses and 23.2% receiving two doses. Age was a key factor (p=0.000) for vaccination acceptance (VA), with those aged 25-45 years having a higher VA than the nonvaccinated (NonVA) (62% vs. 24.7%). VA was lower among those aged 46-65 years (30% vs. 61.3%) and those over 65 years (8% vs. 14%). Lack of awareness of the benefits and misconceptions about the side effects of vaccination are common among different age groups. Males were predominant in our study, with VA higher than NonVA (86% vs. 81.7%, p=0.5), though the difference was not significant. VA by race showed marginal significance (p=0.1), with Whites having the highest VA (40%), followed by Blacks (32%) and Hispanics (20%). Multivariate regression revealed that the 46-65 age group had 80% lower odds of VA compared to the 25-45 group (aOR 0.2, p=0.000), while >65 years had 78% lower odds (aOR 0.22, p=0.021). This regression analysis was adjusted for race (White=Ref, Black p=0.7, Hispanic p=0.1). Upon further stratification of the Hispanic population, VA in the 25-45 years was 6.4% for Black Hispanics and 22.6% for White Hispanics (p=0.000).

**Figure:**
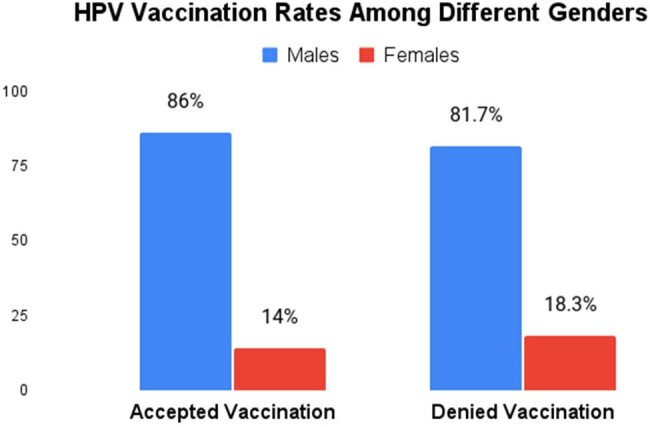

**Conclusion:**

Our study highlights that age and ethnicity are significant predictors of vaccination acceptance, with younger individuals and Whites demonstrating a higher likelihood of vaccination acceptance. Among the racial minorities, vaccination acceptance was lowest in Hispanics. These findings underscore the importance of demographic and cultural factors affecting vaccination rates. Targeted interventions to increase awareness and future studies are needed to determine the factors associated with vaccine hesitancy and work toward implementing new strategies to improve it.

**Figure:**
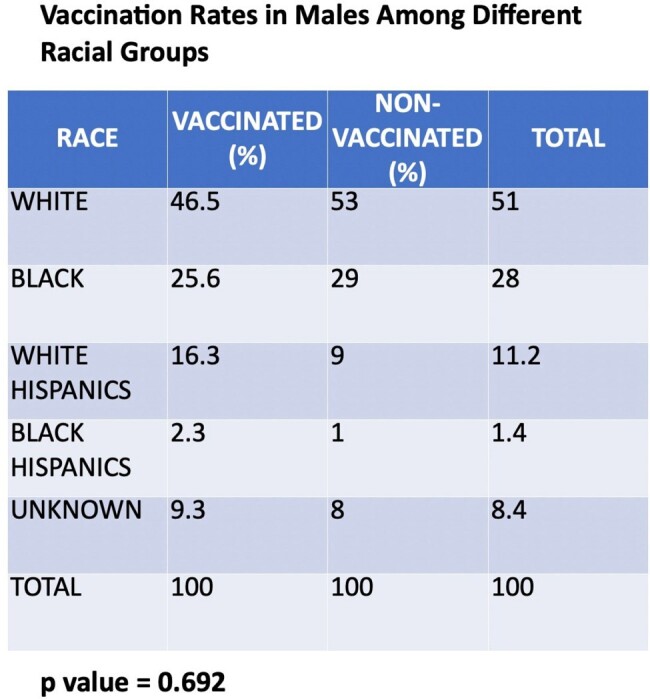

**Disclosures:**

**All Authors**: No reported disclosures

